# Probation and COVID-19: Lessons learned to improve health-related practice

**DOI:** 10.1177/02645505221087980

**Published:** 2022-06

**Authors:** Coral Sirdfield, Helen Nichols, Philip Mullen

**Affiliations:** University of Lincoln, UK; University of Lincoln, UK; Revolving Doors Agency, UK

**Keywords:** probation, Covid-19, pandemic, health, criminal justice system, rehabilitation, qualitative research

## Abstract

Probation staff perform a health-related role involving identifying health-related drivers of offending behaviour; facilitating access to support for these, including continuity of care for people leaving prison; and advising the courts on appropriate sentencing. This study analyses data from probation staff surveys and interviews with people that were under probation supervision during the pandemic to investigate the impact of the response to the pandemic on a) this health-related role, b) the lived experience of accessing health support whilst engaging with probation, and c) partnership working and pathways into healthcare for people under probation supervision.

## Background

**Figure 1.**
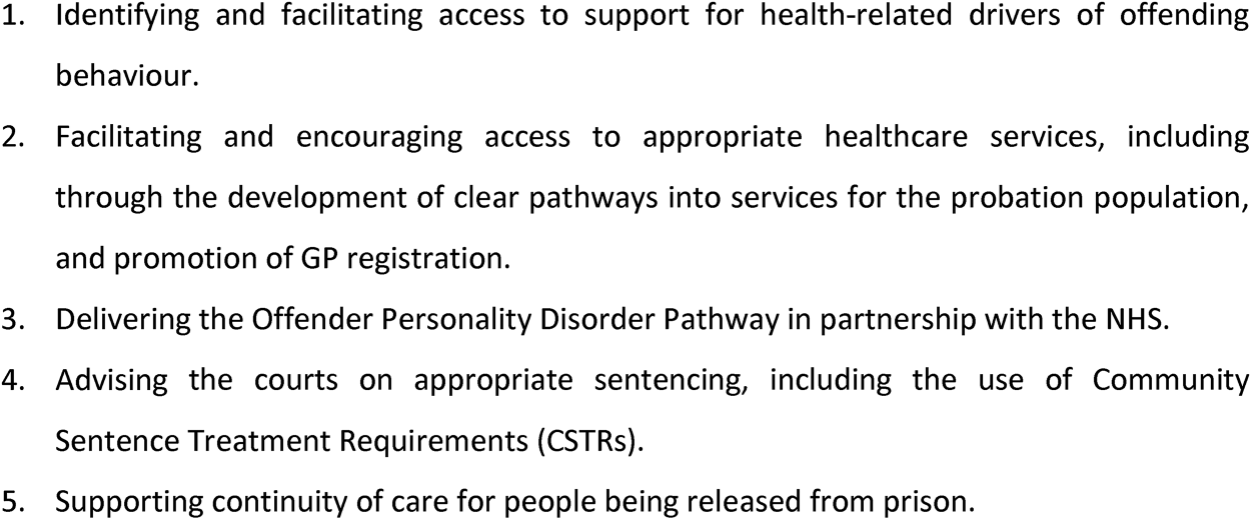
Summary of probation's health-related role.

The National Probation Service (NPS) Health and Social Care Strategy 2019–2022 details a health-related role for probation staff that is summarised in Figure 1 (HMPPS and NPS, 2019). Much of this role is performed in partnership with other agencies, with the exact nature of partnerships varying across probation regions. NHS England is currently piloting a ‘care after custody’ service called ‘RECONNECT’ to improve continuity of care for prison leavers. NPS staff are expected to engage with this service (HMPPS and NPS, 2019; NHS England, 2019). Community Rehabilitation Company (CRC) staff performed a similar role before the reunification of probation.

When compared to the general population, people under probation supervision have relatively poor health with high rates of drug and alcohol misuse, mental illness, suicide and suicidal ideation and smoking (Brooker et al., 2012; Geelan et al., 2000; Mair and May, 1997; HMPPS and NPS, 2019; Newbury-Birch et al., 2009; Pari et al., 2012; Phillips et al., 2018; Sirdifield, 2012). A health needs assessment of 183 people on probation in England reported that:

‘almost half of the sample were identified at risk of alcohol abuse or dependence while 39 per cent…was at risk of substance misuse. Over four-fifths (83%) of the sample smoked tobacco and 13 per cent had been treated for a sexually transmitted infection; all these rates markedly exceed those found amongst the general population’ (Brooker et al., 2009: 49).

Weighted prevalence figures from a study of a stratified random sample of people on probation in one region of England showed that almost 39% had a current mental illness (Brooker et al., 2012). Here, the overall rate of personality disorder was 47.4% compared to 13.7% in the general population (McManus et al., 2016). The rate of suicide amongst people under supervision is 8.67 times higher than that in the general population (Philips et al., 2018). Improving this population's health would not only benefit the individuals concerned, but also produce wider benefits including a reduction in health inequalities, reoffending, and avoidable use of A&E; and improved relationships, compliance and engagement with probation (Revolving Doors Agency, 2017). Consequently, it is vital that despite the pandemic, probation staff can continue to perform their health-related role alongside healthcare partners.

Health and justice partnerships and, in turn, pathways into healthcare have adapted as agencies have responded to social distancing requirements. From the 24^th^ of March 2020, probation in England and Wales adopted ‘Exceptional Delivery Models’, with traditional supervision largely being replaced by doorstep supervision and contact via telephone or other digital platforms. There were concerns about the potential impact of this (House of Commons Justice Committee, 2020), and about reduced access to support services. A thematic review of the arrangements by Her Majesty's Inspectorate of Probation (HMIP) noted that:

‘lockdown has led to a reduction in a number of support services that probation relies on, including mental health and drug and alcohol provision’ (HMIP, 2020 : 4).

However, this review also highlighted the potential opportunity to learn from the adaptations that have been made during the pandemic to improve probation practice (HMIP, 2020; HMPPS and NPS, 2020).

This study aimed to better understand:

1. The practice changes and innovations that have occurred and the impacts of these on probation's health-related work from the perspectives of probation staff and people with lived experience of probation supervision.
2. The lived experience of accessing health support whilst engaging with probation, both pre-pandemic and during the pandemic.
3. The impact of practice changes on partnership working and pathways into healthcare for people under supervision.
4. How learning could improve health-related practice and relationships between probation officers and people being supervised as we emerge from the pandemic.

## Methods

The research was based on analysis of data from 27 qualitative surveys and two follow-up conversations with frontline probation staff across England about the impact of the response to the pandemic on probation's health-related practice, and pathways into care for people under supervision; and 11 semi-structured interviews with people who were under supervision during the pandemic exploring their experiences of accessing healthcare and/or support to manage or improve their health, including through court-mandated CSTRs; and their perception of changes to practice in probation, including partnership working.

Ethical approval was granted by Her Majesty's Prison and Probation Service (HMPPS) National Research Committee and the University of Lincoln; and permission to conduct the research was provided by NPS regional and CRC gatekeepers. All participants provided written informed consent.

### Surveys and follow-up conversations with frontline probation staff

All twelve NPS regions and one CRC^1^ were invited to participate in the research by identifying two to three frontline staff and a senior probation officer to complete the survey. Recruitment was led by key stakeholders in these regions. Data were anonymised, entered into NVivo, and analysed using thematic analysis by a multi-disciplinary team consisting of academics (CS and HN), alongside staff (PM) and peer researchers from Revolving Doors Agency (RDA). This approach was selected as it is suited to topics about which relatively little is known, and to a co-produced analysis that aims to inform policy (Braun and Clarke, 2006). Co-analysing the data enabled the team to view what was being said from a variety of perspectives, ensuring that themes were clearly defined and exploring the transferability of the findings. After independent data review and group discussion, an initial list of 20 codes was agreed and applied to the full dataset. Themes were iteratively refined, and organised into broader themes following further discussion, and an initial thematic map was produced.

The initial findings and thematic map were shared in a workshop attended by NPS staff. Representatives from NHS England and survey participants that were unable to attend received a link to the presentation to watch at their convenience.^2^ Feedback was sought from participants about the accuracy of data interpretation and where further detail should be obtained. Follow-up conversations were held with two survey participants, and findings were discussed at the NPS national health leads meeting. A final thematic map was then produced.

### Interviews with people under supervision

Probation staff were asked to identify two people who had been supervised during the pandemic and had sought help around a health need. To address the potential for selection bias, and for potential participants to feel coerced into participating, areas were asked to recruit an individual who was perceived to have had a positive experience of managing or improving their health, and an individual who was perceived to have had a more negative experience. Staff were asked to emphasise that participation was voluntary, and that participation or non-participation would not influence the relationship with probation in any way. This was reinforced in the participant information resources, and by the researchers prior to commencing the interview proper. Recruitment through this method proved challenging. Consequently, permission was sought and granted to recruit further participants through Revolving Doors’ lived experience membership.

Interviews were conducted largely by telephone (though the option of an online video call was also available) by HN or PM. These interviews were based on a topic guide co-produced with the peer researchers, and were recorded, transcribed verbatim, anonymised, and analysed in Nvivo and through group discussion in the same way as the survey data.

## Research findings

**Figure 2.**
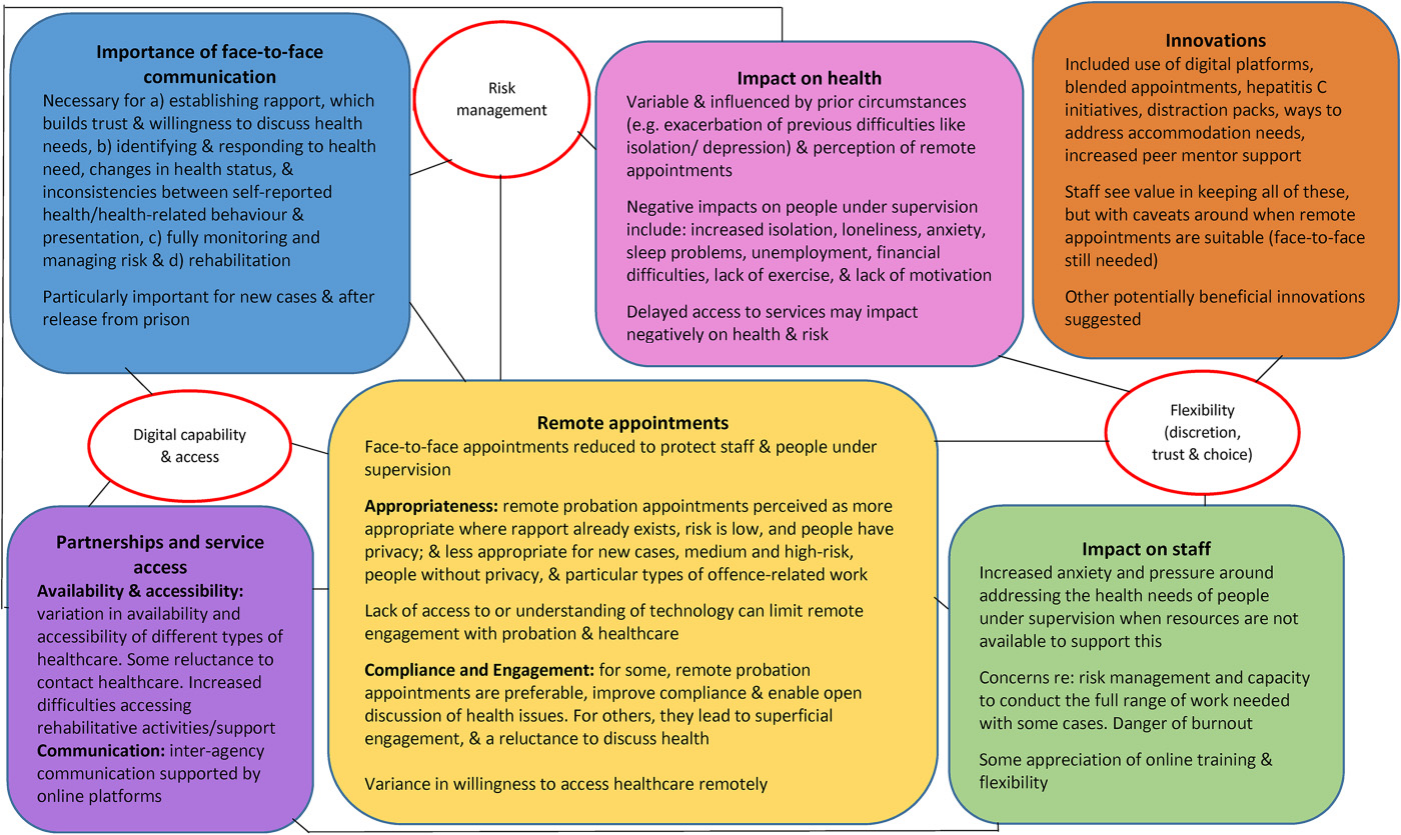
Thematic map from staff survey data.

**Figure 3.**
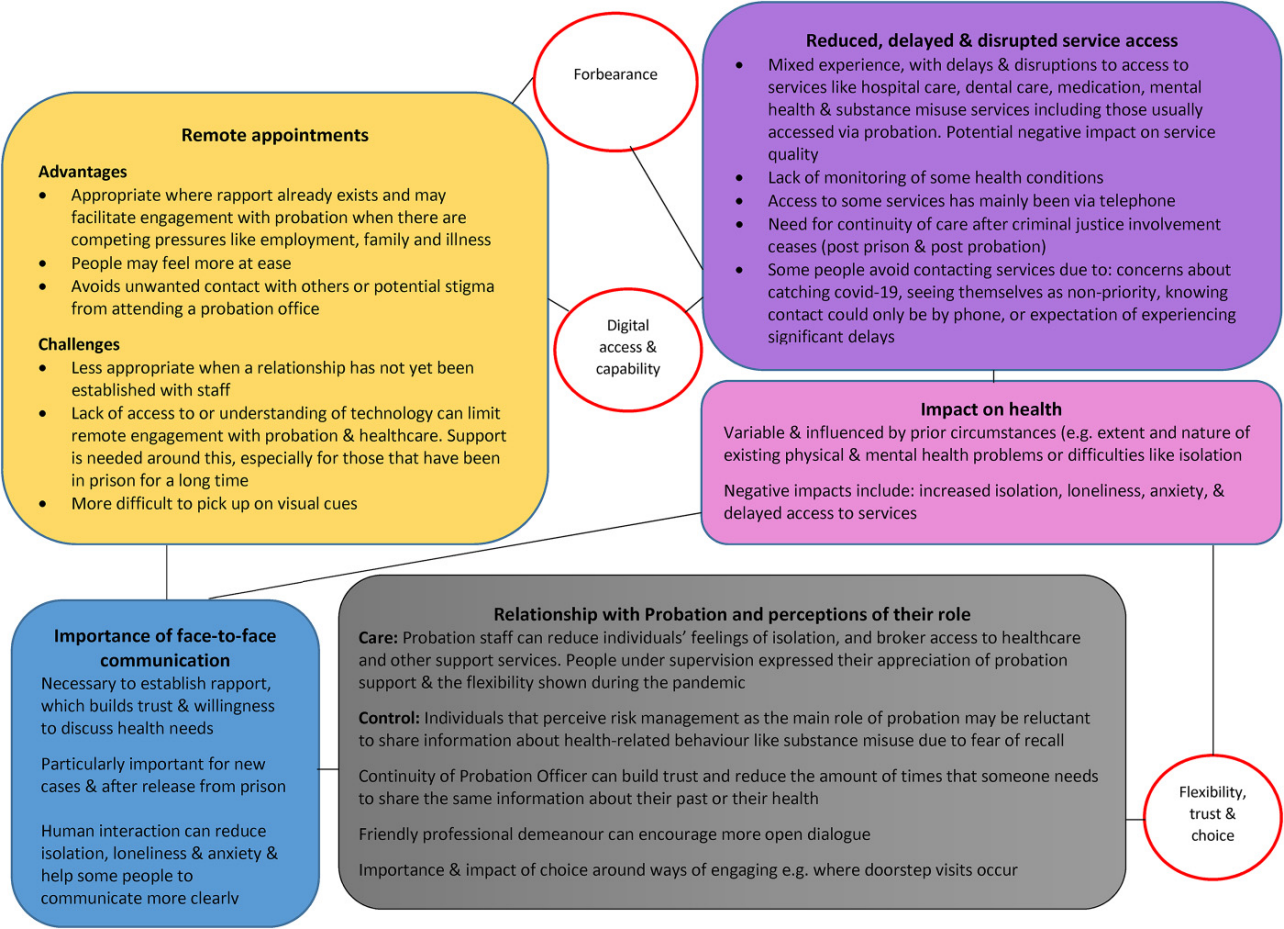
Thematic map from interview data.

Nine themes were generated from the survey data: 1) the importance of face-to-face communication, 2) remote appointments, 3) digital capability and access, 4) risk management, 5) partnerships and service access, 6) health impact, 7) impact on staff, 8) flexibility, discretion, trust, and choice, and 9) innovations. Figure 2 provides a thematic overview of the findings. Eight themes were generated from the interview data (see Figure 3): 1) remote appointments, 2) importance of face-to-face communication, 3) reduced, delayed, and disrupted service access, 4) impact on health, 5) relationship with probation and perceptions of their role, 6) forbearance, 7) digital access and capability, and 8) flexibility, trust, and choice.

### The importance of face-to-face communication

Research demonstrates the influence of pro-social relationships between probation staff and supervised individuals on outcomes such as perceptions of probation, and desistance (Burnett and McNeill, 2005; Barry, 2007; DeLude et al., 2012; Hart and Collins, 2014). Recent research reported that:

‘the quality of the relationship between officer and client is a critical ingredient in achieving better mental health and criminal justice outcomes.’ (Epperson et al., 2020: 725)

Developing good quality relationships remotely can be challenging. Previous research reported that when using video-link technology to contact people in prison, probation staff found that ‘the screen made it difficult to create rapport with offenders, something that was seen as critical to creating a constructive worker-client relationship’ (Phillips, 2017: 216). Concerns were also previously expressed around the use of remote kiosks and telephone supervision by CRCs:

‘kiosk meetings are never likely to be appropriate…telephone supervision should only be used in exceptional circumstances and not in isolation’ (House of Commons Justice Committee, 2018: 5).

During the pandemic, face-to-face contact between probation staff and supervised individuals was necessarily reduced to prevent transmission of Covid-19, a change of practice that may be seen negatively considering the above. Survey responses indicated that staff perceived face-to-face appointments as essential for high and medium-risk cases, to establish rapport with new cases, and to support open and honest discussion of health needs:

‘For service users for whom face to face contact is important to build relationship it has been hard. This is particularly true when taking on a new service user.’ (Staff 1)

‘Maintaining a good and open relationship with service users has been more challenging than I have experienced before…service users have found it challenging to open-up about health and mental health issues and disclose when they are struggling or need support.’ (Staff 17)

Similarly, participants under supervision felt that face-to-face communication was important to establish rapport, and through this, support people to feel more comfortable discussing health issues. It could also reduce isolation, loneliness and anxiety, and some people found it easier to communicate face-to-face than over the telephone or other digital media:

‘It's just not being able to see them that is the frustrating bit. It just makes you a bit more anxious, a bit more lonely.’ (Interviewee 3)

‘I do like to be able to see someone face to face. I think you can get your point across better, can’t you…especially if you’re meeting someone for the first time.’ (Interviewee 9)

Staff considered face-to-face contact as key for assessing visual cues to support their role in identifying and facilitating access to support for health-related drivers of offending behaviour, and monitoring and managing risk, including detecting any mismatch between self-reported and observable signs of health-related behaviour (e.g. substance misuse):

‘A lack of face-to-face contact not only restricts what it is possible to observe in terms of health-related challenges but also our ability to interact in person about these challenges and come up with solutions/plans.’ (Staff 1)

‘Trying to talk about thoughts and feelings or do work with people is more difficult when you cannot see their body language or facial expressions, which can be telling a different story to what their words are telling us.’ (Staff 24)

### Remote appointments

Despite the above concerns, remote appointments were perceived as preferable in some circumstances, and as having the potential to improve compliance and engagement with probation for some people, including around health issues. However, probation staff also had concerns remote appointments could lead to superficial engagement and prevent discussion of health issues:

‘For some service users virtual meetings have been easier for them, in particular if their mobility is affected or if they live some distance from town/offices etc. Some service users feel more comfortable having discussions with professionals whilst sitting in their own familiar surroundings rather than in a sterile office.’ (Staff 13)

‘It is easier for them to answer calls during periods of health difficulties, whereas comparably they may have failed to attend the office.’ (Staff 14)

‘Phone call appointments are harder to engage them with and are often forgotten or overlooked as they aren’t seen as a ‘proper’ appointment with probation. This has meant that discussing topics such as health and mental health is harder over the phone.’ (Staff 17)

Staff and people under supervision perceived remote probation appointments as more appropriate where rapport had already been established between staff and supervisees. Staff also regarded them as more appropriate where risk had been assessed to be low, and the individual had a private space in which to talk; whilst people under supervision said remote appointments enabled them to better manage competing pressures like employment, family, and illness; save travel time and costs; and avoid unwanted contact with others, or potential stigma from attending a probation office. Some participants on probation reported that it had changed their perception of probation to being supportive, rather than purely about surveillance:

‘I’ve bumped into people that I was in prison with that I didn’t particularly like… when you go it’s a probation office – you might as well put a sign on your head saying I’m here because I’m a criminal…Whereas at this moment it’s just somebody turning up, it could be my friend…it just felt like I’m talking to a friend, it didn’t feel like I’m being judged.’ (Interviewee 10)

Remote appointments were perceived as less appropriate for undertaking particular kinds of offence-related work, for example around domestic violence, and when individuals lacked the digital capability and/or access to engage in this way and made it difficult for supervisees to recognise visual cues from staff. Some supervisees felt that remote contact with health services was lower quality:

‘For some service users who are in abusive relationships, it has not been appropriate to deliver healthy relationship work remotely…In addition, with the Mental Health Treatment Requirement, some female service users have not felt comfortable discussing their mental health whilst at home around family members.’ (Staff 3)

‘I do not believe that the remote management of high and medium risk offenders is a sustainable practice.’ (Staff 5)

‘I've done CBT (Cognitive Behavioural Therapy) before in face-to-face capacity but trying to do it over the internet on video calls, it's really very different…You get instances where your sound drops out… you have to ask them to repeat, and every now and again the screen freezes and you drop out of the conversation.’ (Interviewee 1)

Many health services also (largely) replaced face-to-face contact with alternatives during the pandemic. Staff perceived the response of supervised individuals to this as mixed.

### Digital capability and access

Engagement with services, including probation, can be limited by a lack of access to technology (for example not owning or being able to access smart technology), and/or a lack of understanding of how to use this technology. Consequently, this ‘digital divide’ should inform decisions regarding the appropriateness of remote rather than face-to-face probation and healthcare appointments. Currently, people in prison may be released into a society where they are excluded from accessing information and services (for example to gain employment, information about health, or to support self-care and engagement with online support), due to a lack of digital skills (Reisdorf and Rikhard, 2018). Whilst efforts were made by probation to supply mobile phones where possible, these did not always support video-calls, and people may also face limitations on technology use due to licence conditions. This raises questions around how to prevent this digital divide from widening.

Whilst some people under supervision were confident using technology, others shared challenges that they and others encountered around accessing and understanding technology. There is a need for support to address this digital divide, particularly for people that have been in prison for an extended period:

‘I had never used the computer, ever in my life; and guess what, everything is done by computer.’ (Interviewee 2)

‘It was a good job that the hostel was there because we had to do a video link, and I'm not good with technology, so they had to set it all up for me; and as soon as they saw my abscess, they brought me in; they gave me antibiotics and it went.’ (Interviewee 3)

### Risk management

Staff viewed face-to-face contact as particularly important for medium and high-risk cases and as an essential part of fully monitoring and managing risk in relation to health-related behaviours, such as identifying substance misuse or changes in mental health that may indicate increased risk to self or others. Staff also expressed concerns that delays in access to, or the absence of, healthcare services may impact negatively on risk.

### Service access

Two themes were identified regarding service access (‘partnerships and service access’ and ‘reduced, delayed, and disrupted service access’). The survey and interview data suggested that the pandemic's impact on the availability and accessibility of healthcare services was variable. Whilst some services were unaffected, access to others was difficult or delayed, including mental health and substance misuse services, social care assessments, GPs, dentists, and hospitals. Whilst some people under supervision described straightforward access to medication, others had experienced slight delays. Many reported difficulties in accessing alternatives to medication for mental ill-health. Participants also spoke about reduced access to primary care and reduced or no monitoring by professionals of their health conditions:

‘Before the pandemic came, I was having quite a lot of surgery on my legs because of my arteries and stuff…as soon as the pandemic came in, appointments stopped… I've got more white cells than red cells…but since I've come out in the pandemic, I've not had any monitoring.’ (Interviewee 2)

‘We got in touch with MIND (Mental Health Charity) as well, you’ve heard of MIND, and again we’ve heard nothing because of the fact it’s COVID.’ (Interviewee 8)

‘Getting my medication, there's been a couple of times where it's been due, and it hasn't turned up until the day later.’ (Interviewee 1)

‘Interventions provided by partners have generally suffered…Assessments, by social care for instance, are generally done remotely. Home visits and in person visits have reduced from partners. The communication between agencies has generally been good but the work done with service users has suffered.’ (Staff 8)

‘I have several cases who have had operations cancelled. One in particular is recovering from bowel cancer. He is unable to drive professionally…until he has had one final test/scan, which he has been waiting nine months for. Another is waiting for an operation to change his pacemaker…Another needs to be sectioned due to his paranoid schizophrenia worsening, his consultant agrees but stated that at the moment the spaces are limited.’ (Staff 10)

People on probation encounter many barriers to accessing healthcare services (Sirdifield et al., 2019), and have faced additional barriers during the pandemic due to the digital divide, and a perception that they should avoid contacting services to avoid overburdening them:

‘One of my cases is not going to the doctors as he knows they are strained and that things may be delayed due to the pandemic. Another…has increased anxiety…and is reluctant to call the doctors.’ (Staff 19)

In some regions, NHS healthcare staff had previously provided interventions in probation offices, but this had ceased during the pandemic. Similarly, CSTRs were perceived as negatively impacted as drug and alcohol testing were greatly reduced. Many unpaid work placements ceased, resulting in people being unable to complete the requirements of their sentence. Adaptations were made where possible, such as offering one-to-one interventions rather than group work. However, this placed additional burdens on staff, slowed down service delivery, and some questioned the quality of interventions provided.

In some cases, probation staff felt that they needed to bridge the gaps in healthcare support for supervised individuals. People under supervision valued probation’s support, with some expressing a desire for this to continue after their official contact with probation had ceased:

‘I’ve been so depressed and it’s just me having sit back to two years ago when I was locked up and I had a therapist that was helping me and giving me techniques and things to do… it is mentally draining again because it’s just you that you…don’t have anybody else. I feel like there should be a period of time after your probation period where you are still able to access certain things.’ (Interviewee 6)

‘After having a Probation Officer for that length of time, and [Probation Officer] being a bit of a counsellor, well I didn't really want to knock it on the head and say – right, that’s it, probation is over…I'd like to keep talking to somebody.’ (Interviewee 4)

Whilst the pandemic created challenges some positive changes occurred, particularly in partnership working. Inter-agency communication was initially challenging, but was felt to have improved, assisted by platforms like Microsoft Teams (although this did not work for those without a private workspace, or with connectivity issues). Support was also provided by the UK government to help those with accommodation needs. In some cases, access to medication had improved, whilst in others it was problematic:

‘I only have one service user with a treatment requirement – Mental Health. I have found that the CPN (Community Psychiatric Nurse) supporting him has been excellent and we have tried to combine our contact with him to ensure that he is appropriately supported. This has been a really positive outcome of the pandemic as previously my experience of MHTR has not been very integrated.’ (Staff 14)

‘Working with partnership agencies in my experience has in fact improved. Teams meetings has meant it is easier to get people round a table and resolve issues sooner.’ (Staff 17)

‘There has been an advantage in there being a quicker more streamlined process for people to access scripting with drug services.’ (Staff 18)

‘Those rough sleeping or in insecure accommodation have been most impacted by the pandemic and response in terms of being offered accommodation.’ (Staff 4)

‘Scripts are being picked up at chemists and taken without supervision. One offender admitted that he now has a stash of methadone from attempting to reduce on his own.’ (Staff 5)

### Health impacts

The impact on the health and welfare of supervised individuals was regarded as largely negative with survey and interview data describing increased isolation, loneliness, anxiety, sleep problems, loss of employment, financial difficulties, lack of exercise, delayed access to services, and lack of motivation. The extent of the impact was affected by individuals’ circumstances prior to the pandemic (i.e. whether they had any existing health problems or experienced difficulties such as isolation), the extent to which their care was disrupted or delayed, and their willingness and ability to access services remotely.

### Impacts on staff

Probation staff have adapted to new ways of working and engaging with healthcare partners and supervised individuals, including working from home, increasing their digital capability, and managing pressures from workload and personal circumstances (such as home schooling). Probation is familiar with change, and research suggests that despite a changing political discourse, staff remain motivated by the desire to work with people, and maintain probation values that are ‘inclusive of non-judgemental attitudes towards offending, a belief in offenders’ capacity to change, and recognition of socio-structural disadvantage as determinant of offending behaviour’ ((Tidmarsh, 2020: 3) See also: Annison et al., 2008; Farrow, 2004; Willis, 1986). A study in a CRC states that ‘a willingness to react to crises, to drop everything to ensure a client's wellbeing, demonstrates how practitioners prioritise working with ‘people’ over working with ‘things’’ (Tidmarsh, 2020: 6), but warns that practitioners’ professional values can be used to justify increased workloads, which may exacerbate existing problems around staff sickness and work-related stress. These values and concerns were apparent in the survey responses, where a participant reported:

‘A sense of helplessness amongst probation staff as the resources usually available to deal with health issues are severely hampered by Covid restrictions and often the probation officer managing the case is the only professional having any sort of meaningful contact with the service user. This can cause the probation officer to feel overwhelmed and anxious that they are not able to get the right sort of resource for that person.’ (Staff 13)

Staff undertook additional work to bridge gaps in service provision, but still felt unable to fully measure and monitor risk or undertake the full range of work that was required with some individuals. Continuation of these pressures could lead to burnout – a concern recognised by one participant:

‘Staff are working harder and longer than ever before, and burnout/mental health/wellbeing concerns are a key worry for middle managers such as myself.’ (Staff 22)

### Flexibility, discretion, trust and choice

Under the Labour government that pledged to be ‘tough on crime and tough on the causes of crime’, and the Conservative government that presided over *Transforming Rehabilitation*, probation practice was increasingly standardised through a managerial/target-driven occupational culture, with staff autonomy and discretion being reduced. Policy emphasised ‘control’ (protection of the public, punishment, and risk management) rather than ‘care’ (rehabilitation) (McWilliams, 1986; Phillips, 2011; Tidmarsh, 2020; Whitehead and Statham, 2006). However, during the pandemic staff have benefited from the ability to work flexibly, and use discretion in supervision:

‘We’ve been able to be more flexible in approach to supervision and tailor this more to the service users’ needs – when there has been more emphasis on professional judgement, rather than blanket rules based on risk levels.’ (Staff 14)

‘Having a combination of office and home-based work and face-to-face, phone calls and doorstep visits supervision has worked well…in terms of managing my caseload and helping me through the pandemic.’ (Staff 6)

People under supervision appreciated the flexibility shown by probation and other services including staff delaying reducing a level of risk categorisation to ensure that an individual could maintain some face-to-face contact and ensuring that doorstep visits were conducted in a neighbouring street rather than outside a home to avoid any potential stigma if neighbours overheard the conversation. Blended supervision was perceived to work well in some circumstances, particularly when the supervised individual was involved in decisions around striking the right balance of communication.

### Innovations

**Figure 4.**
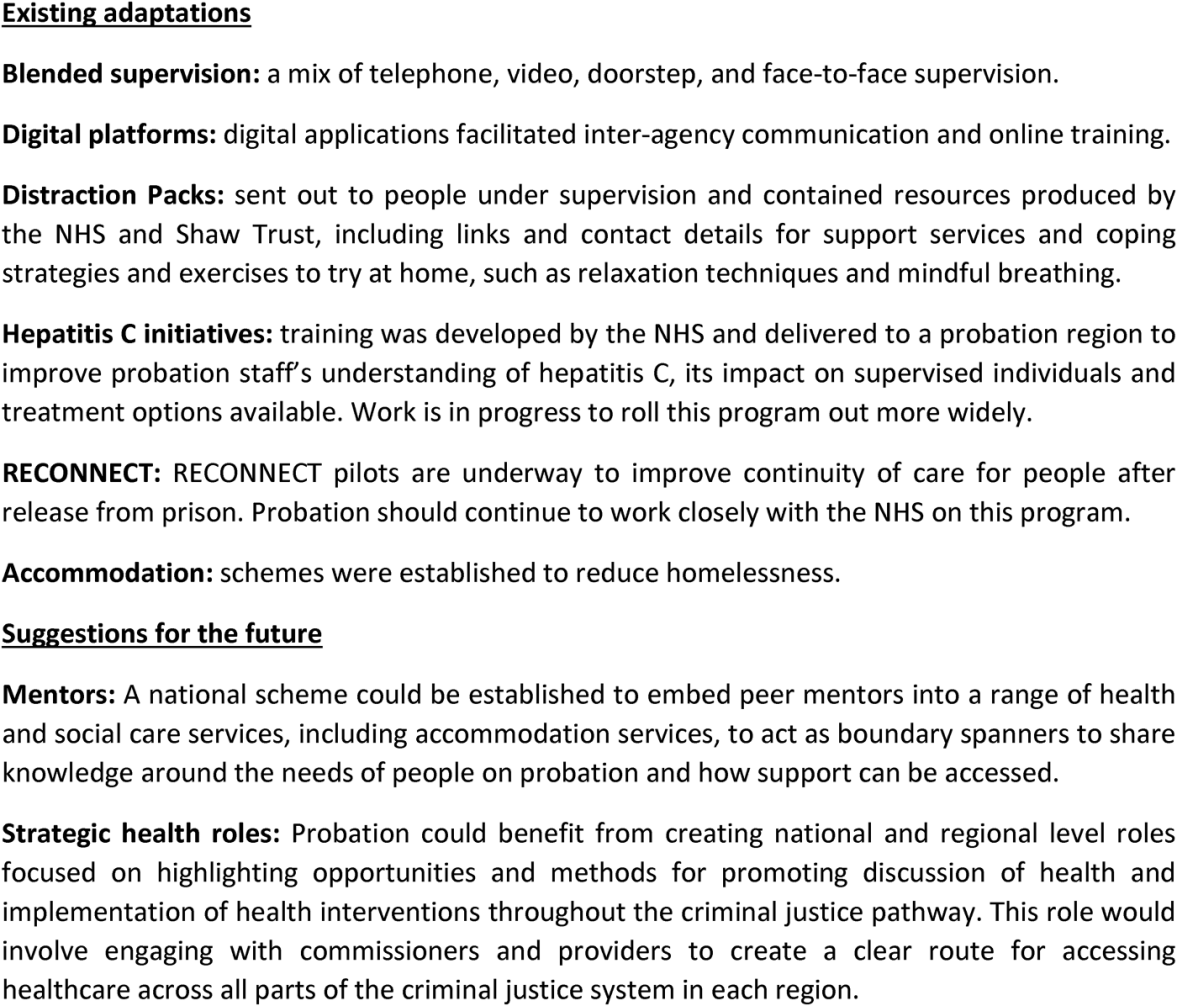
Innovations.

Innovations have occurred because of the pandemic (see Figure 4). Staff saw value in keeping all of these, although remote appointments with both probation and healthcare partners are perceived as more suitable in some circumstances than in others. Staff also suggested additional innovations that could be trialled in the future.

### Forbearance

Supervised individuals demonstrated forbearance in their attitude towards the changes in how probation and health services could be accessed, and any difficulties experienced. Some individuals avoided contacting services because they did not see themselves as a priority and did not wish to place undue burden on services. Participants also appreciated the measures employed to protect their health and to enable them to continue to engage with probation:

‘I've had to wait for unfortunately for over 25 weeks now for an appointment at the hospital; but given the circumstances and their specialist catching COVID, it's understandable.’ (Interviewee 1)

‘I'd already met up with the pandemic while I was inside… I also had to give the staff their due. The wing that I was on was all insulated, nobody came through, we wasn't contacted with the outside world…and we were kept safe. I know it takes a lot of organisation.’ (Interviewee 2)

### Relationship with probation and perceptions of their role

Some participants stated that contact with probation had helped to reduce feelings of isolation, and shared examples of how probation staff had supported them to gain access to healthcare and other support services. Others perceived probation's role to be primarily focused on risk management, and consequently, were reluctant to be open and honest about their health due to fears that this may lead to reimprisonment:

‘I think health care and probation have gone out of their way to help me, even though I struggled because of not being able to see them face to face as much as I normally do.’ (Interviewee 8)

‘In prison…there is always somebody you can go to. But with the stigma that surrounds probation not many people want to open up to them because you feel like ‘I’m at the risk of taking drugs again’ or whatever it may be, you get that ‘oh’ I might get recalled, I’m definitely not saying something.’ (Interviewee 6)

Willingness to discuss health was also affected by the development of a relationship with probation over time, which may be positively influenced by staff having a friendly professional demeanour:

Interviewer: ‘Is there anything in particular that has supported you to feel that you can talk to them honestly?

Participant: I suppose, it might sound a bit corny but, it’s not judgmental, that enables us to open up initially and I genuinely got the impression that she cared, and she wanted to help…when I got out of prison originally, I wasn’t speaking like this.’ (Interviewee 5)

‘The probation officer that I’ve got at the moment, if she rings me, she can tell if I’m a little bit down, she’s already got the measure of me…So to build a relationship with another one, that all takes time…it’s that… gut wrenching feeling of having to just tell the whole story again.’ (Interviewee 9)

## Discussion

This research aimed to increase understanding of the changes and innovations probation made in response to the pandemic, the lived experience of accessing health support whilst under supervision, and the impact of these on probation's health-related work and partnership working and pathways into healthcare for supervised individuals. As a qualitative study, findings may not be generalisable/transferable beyond those taking part. We did not collect demographic information about study participants, but future research may wish to do so to compare experiences across different groups or settings. The findings presented here provide in-depth insights into the experiences and perceptions of both staff and those under supervision which both add to and echo themes within the wider probation literature.

Perhaps the most significant change has been the reduction of face-to-face contact with people under supervision. In some cases, this was perceived as impacting negatively on staff's ability to identify and facilitate access to support for health-related drivers of offending behaviour and monitoring and managing risk in relation to this - suggesting a need to return to face-to-face supervision. However, whilst it is undesirable for remote appointments to replace *all* face-to-face contact long-term, findings largely reflect those from a recent study in three CRCs (Dominey et al., 2021), suggesting that remote appointments could usefully complement traditional practice in some circumstances. The relationship between probation staff and people under supervision remains key to achieving good health and criminal justice outcomes. Increasing the amount of discretion that probation staff have, and offering flexibility, trust, and choice to those under supervision about how they wish to engage once rapport has been established, may positively influence this relationship – leading supervisees to view probation as largely supportive rather than surveillance focused.

Guidance around blended supervision has been developed by HMPPS specifying that face-to-face contact should occur every four weeks as a minimum, and an outcomes study of blended supervision has also been proposed. Current national standards state that ‘methods of contact will differ from person to person and will take account of risk and need’ (HMPPS, 2021: 9). Similarly, findings from this study suggest that rather than a prescriptive ‘one size fits all’ model of supervision, a more flexible approach to the use of blended supervision is required. There are several factors to consider when deciding whether to offer remote appointments to complement traditional practice:

- **Risk:** face-to-face contact is needed in *all* cases, but staff viewed it as more important for those in high and medium risk categories.
- **Digital capacity and capability:** digital exclusion is an issue for some people under supervision (HMIP, 2020), and this can be further impacted by licence conditions and a lack of funds to purchase and maintain appropriate technology.
- **Privacy:** it is essential that people have access to a private space to support them to speak more openly about their needs during remote appointments.
- **Rapport:** Remote supervision may work better when rapport has already been established and may not be as suitable at the start of a new relationship.
- **Identified health needs:** face-to-face contact is key for identifying and monitoring changes in health status.
- **Individual preferences and circumstances:** the option of remote appointments may be preferable to save on travel time and expenses; to support people to attend probation when experiencing competing pressures; and to avoid unwanted contact with others or stigma from attending a probation office. Others may benefit from face-to-face appointments to enable them to read visual cues and articulate health needs more easily. Taking individuals’ preferences into account strengthens engagement and supports desistance.
- **Quality of engagement:** whilst some may engage well in remote appointments, others may engage superficially or struggle to articulate health needs.
- **Nature of work:** remote appointments may not be appropriate for some types of work, for example around domestic violence.

If access to probation and health services supporting rehabilitation is going to be increasingly dependent on the use of technology in the future, then it is essential that steps are taken to avoid widening the digital divide that some individuals experience. Provision of digital skills training pre-release and in the community and providing means for people to access technology who could otherwise not afford it will be key.

The pandemic has also impacted negatively on the roles of probation staff in facilitating access to appropriate healthcare services, including through CSTRs, and in supporting continuity of care after release from prison, as in some instances access to care has been delayed, disrupted, or only possible through online or telephone appointments. The experiences of supervised individuals in this study reflected findings of earlier research, showing a negative impact on their health and wellbeing (Carr, 2021; Revolving Doors Agency, 2020). Probation staff have felt pressured to bridge the gap in service access, leading to concerns about burnout. It is important that staff can access practical and emotional support through supervision and wellbeing services to reduce the likelihood of this.

Finally, there have been some beneficial impacts of the response to the pandemic, including improved inter-agency communication using online platforms, increased flexibility and discretion in probation practice, and an increased focus on meeting accommodation needs. Continuation and evaluation of these innovations may be useful. Similarly, probation staff suggested potential future innovations which could be piloted and evaluated. For example, the introduction of a strategic health role could support staff in facilitating access to services and reduce some of the pressures that they have experienced around this.

## Conclusion

The response to the pandemic has led to improved inter-agency communication, increased flexibility, and a focus on meeting accommodation needs. However, many aspects of probation's health-related role have been made more difficult during the pandemic. Identification and monitoring of health needs during remote supervision is challenging. The health of those under supervision has often worsened and accessing health support for many has been made more difficult, with access often reliant on an individual's digital capacity and capability, and probation staff feeling a need to bridge gaps in service provision. There is a clear need to address the digital divide, and to support staff to prevent burnout. Remote supervision is unlikely to be helpful if used alone but may be beneficial when employed carefully as part of an individually tailored blended supervision process.

## Notes

1. We wished to include people on probation classified as low-risk and to provide insight into any differences in staff role between CRCs and the NPS. However, as data were being collected during the pandemic we did not invite all CRCs to participate for practical reasons.

2. See https://probation-and-covid19.blogs.lincoln.ac.uk/findings-and-outputs/ for the slides.
